# Malaria and the climate in Karachi: An eight year review

**DOI:** 10.12669/pjms.36.ICON-Suppl.1712

**Published:** 2020-01

**Authors:** Fivzia Herekar, Sundus Iftikhar, Ahsana Nazish, Sabeen Rehman

**Affiliations:** 1 Fivzia Herekar, FCPS (Infectious Diseases), Department of Internal Medicine, Indus Hospital Research Center, The Indus Hospital, Karachi, Pakistan; 2 Sundus Iftikhar, Mphil (Statistics), Indus Hospital Research Center, The Indus Hospital, Karachi, Pakistan; 3 Ahsana Nazish, MSc (Health Policy and Management), Malaria Program, Indus Hospital Research Center, The Indus Hospital, Karachi, Pakistan; 4 Sabeen Rehman, FCPS (Internal Medicine), Department of Internal Medicine, Indus Hospital Research Center, The Indus Hospital, Karachi, Pakistan

**Keywords:** Anopheles, Malaria, Seasonality

## Abstract

**Background and Objective:**

Malaria is an arthropod-borne infectious disease transmitted by the mosquito Anopheles and claims millions of lives globally every year. Reasons for failure to eradicate this disease are multifactorial. The seasonality of the malaria is principally determined by climatic factors conducive for breeding of the vector. We aimed to study the relationship between climatic variability and the seasonality of malaria over an eight-year duration.

**Methods:**

This was a retrospective medical chart review of 8,844 confirmed cases of malaria which presented to The Indus Hospital, Karachi from January 2008 to November 2015. Cases were plotted against meteorological data for Karachi to elicit monthly variation.

**Results:**

A secular incline and seasonality in malaria cases over the duration of eight years was seen. More cases were reported in the summer, rainy season compared with the other three seasons in each year. There was significant association with specific climate variables such as temperature, moisture, and humidity.

**Conclusion:**

There is a marked seasonal variation of malaria in Karachi, influenced by various environmental factors. Identification of the ‘the concentrated period’ of malaria can be helpful for policymakers to deploy malaria control interventions.

## INTRODUCTION

Over one billion vector-borne diseases occur annually, with more than a million deaths from malaria, dengue, chikungunya and yellow fever, making up to 17% of infectious diseases.^1^ Amongst multiple vectors; the most well-known is the mosquito, responsible for malaria along with dengue, chikungunya, yellow fever, zika, West Nile fever and Japanese encephalitis. Malaria is a high burden yet preventable disease. It is still endemic in the WHO regions of Africa, South-East Asia and the Eastern Mediterranean. Despite declining incidence since 2000, cases in 2015 were still a staggering 214 million with estimated deaths of 438000.^2^ One million cases were reported from Pakistan that year despite decreasing trends between 2010 to 2015, when 400,000 fewer cases of malaria annually were seen from previous years.^3^

The malarial parasite requires the female Anopheles mosquito to complete its life cycle before transmission back to the human. There are 430 types of Anopheles mosquito and 30-40 transmit malaria.^4^ Of 38 species identified in Pakistan^5^, Anopheles subpictus, Anopheles culicifaciens, and Anopheles stephensi are most prevalent.^6^ They breed in turbid, waterlogged areas and animal ponds rich in inorganic matter and fauna. Its life cycle is completed within the mosquito in 10-18 days, when the vector must survive in the environment. Environmental factors thus have an important role in transmission. The climatic factors most conducive to survival of the malarial parasite are rainfall, a temperature of 16-18°C and humidity levels of 55-80%.^7^-^10^

Globalization, unplanned urbanization, global warming and agricultural reforms has led to deforestation and irrigation projects, creating ideal breeding sites for mosquitoes. Studying the seasonal peaks of diseases caused by mosquitoes and the impact of climate on their life cycle will help predict and map areas and seasons of high burden. In addition, programmatic steps, such as establishing case definition, diagnosis, steps for treatment and prevention, can be incorporated, targeting seasonal variations of the disease burden to achieve better overall disease control.

## METHODS

A retrospective medical chart review was conducted at Indus Hospital, Karachi (TIH). Appropriate ethical approvals were in place (IRB no. IRD_IRB_2015_08_004). All confirmed cases of malaria presenting to TIH from January 2008 until November 2015 were included in the study. The incidence of malaria was recorded per month and compared to various meteorological indices for the same period. Meteorological data included daily maximum, minimum, and mean temperatures, humidity, dew point, wind speed, and precipitation. Information was downloaded from Weather Underground, where data is crowd-sourced by global environmental sensors and air pollution monitors. Data for the aformentioned variables was accessed from the closest station to TIH, i.e. Jinnah International Airport, Karachi (http://www.wunderground.com/history/airport/OPKC/2015/3/15/DailyHistory.html?&reqdb.zip=&reqdb.magic=&reqdb.wmo=).

### Statistical analysis

Data was analysed using Eviews 9 software. Partial correlation was assessed between malaria cases and climate variables adjusting for years. Cameron and Trivedi test was applied to assess the over-dispersion assumption of Poisson regression. The Negative Binomial regression was used because Poisson regression demonstrated an insignificant P-value (<0.05) despite a negative slope coefficient, demonstrating the latter regression method was inappropriate for this dataset. Pearson correlation was applied to assess the association between various climate variables. In addition, co-integration between variables was assessed using Johnson co-integrated test. Johnson co-integrated test includes trace and maximum Eigen values test to assess co-integration. These two tests reported 5 co-integrated equations showing that the variables were co-integrated.

The lag length for climate variables was selected using lag length criteria using the sequential modified likelihood ratio (LR) test statistic, final prediction error (FPE), Akaike information criterion (AIC), Schwarz information criterion (SC) and Hannan-Quinn information criterion (HQ). However, all the aforementioned criteria indicated different lag lengths. Models on several lag lengths were therefore built (1 to 7-the maximum lag length selected by various lag selection criterion).

All the climate variables with low or no multicollinearity were included in the model. Their lag values were included in the multiple Negative Binomial regression models. The final model was selected based on the Akaike Information Criterion (AIC). The model with minimum AIC and HQ value was chosen as the final model. Model with lag (1) was found to have minimum AIC and HQ values.

## RESULTS

**Fig.1:**
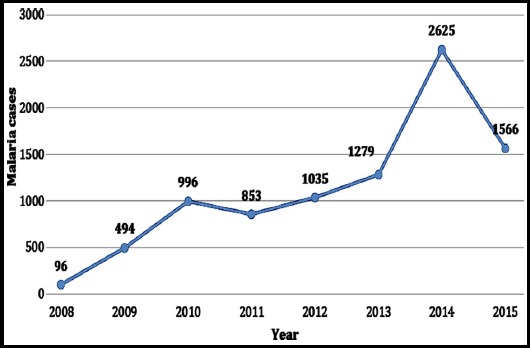
Annual malaria cases at TIH, Pakistan, 2008-2015.

A total of 8,944 malaria cases were reported over eight years with minimum cases reported in 2008 (n=96) and maximum in 2014 (n=2,625, Fig.1), showing a secular incline and seasonality in malaria cases. Of the total cases, 4730 (53%) had species identified and the most prevalent was plasmodium vivax 96.6%, the remainder comprised of mixed infection and Plasmodium falciparum.

**Fig.2:**
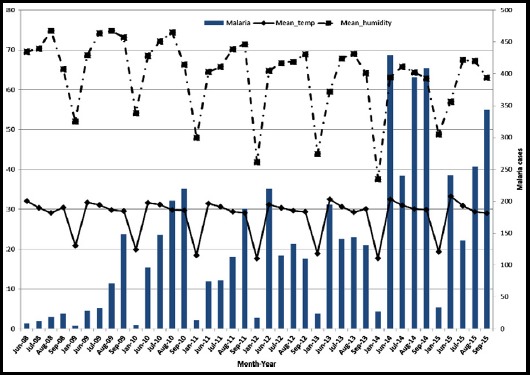
Relation of monthly malaria cases with temperature and humidity.

There are four seasons in Pakistan: a cool, dry winter (December to February), a hot dry spring (March to May), the summer monsoon season (June to September) and the retreating monsoon period (October and November).^11^ Malaria cases were reported more frequently in the summer rainy season as compared to the other three seasons in each year (Fig. 1 and 2, Table-I) with peaks from June to October.

**Table I:** Month-wise distribution of malaria cases at TIH across 2008 to 2015. (SD=standard deviation)

Months	Number of months	Malaria Cases	

		Mean (SD)	Min-max
Jan	8	15.8 (11.9)	0-34
Feb	8	15.6 (12.6)	0-36
Mar	8	29.6 (25.6)	1-74
Apr	8	55.1 (45)	0-136
May	8	119 (127.7)	3-399
Jun	8	161.4 (139.3)	8-429
Jul	8	112.8 (72.4)	12-240
Aug	8	166.1 (117.4)	19-395
Sep	8	196.8 (126.2)	24-409
Oct	8	156.8 (79.5)	16-293
Nov	8	68.6 (47.3)	8-147
Dec	7	23.4 (12.3)	5-40

The maximal humidity in Karachi was in August (86.3%), with a minimal temperature of 32.7°C, leading to a high number of cases in September i.e. with a lag of one month. Malaria cases were significantly associated with various climate variables (Table-II). A substantial positive correlation with mean temperature (0.658, p<0.0001), mean dew point (0.687, p<0.0001) and mean humidity (0.65, p<0.0001) was seen. Moreover, moderately positive correlation was found between mean wind speed (0.583, p<0.05), wind direction degrees (0.516, p<0.05) and cloud cover (0.433, p<0.05). A negative but strong correlation with mean sea level pressure (-0.639, p<0.0001) was also seen.

**Table II:** Partial correlation of malaria cases with climate variables (adjusted for years).

Climate variables	Partial correlation	Climate variables	Partial correlation
Max Temperature (°C)	0.592**	Mean Sea Level Pressure (hPa)	-0.639**
Mean Temperature (°C)	0.658**	Min Sea Level Pressure (hPa)	-0.635**
Min Temperature (°C)	0.672**	Max Visibility (Km)	-0.199
Max Dew Point (°C)	0.674**	Mean Visibility (Km)	0.29
Mean Dew Point (°C)	0.687**	Min Visibility (Km)	0.00
Min Dew point (°C)	0.697**	Max Wind Speed (Km/h)	0.525*
Max Humidity	0.533*	Mean Wind Speed (Km/h)	0.583*
Mean Humidity	0.65**	Max Gust Speed (Km/h)	0.182
Min Humidity	0.642**	Cloud Cover	0.433*
Max Sea Level Pressure (hPa)	-0.644**	Wind Direction Degrees	0.516*

* P-value<0.05,

** P-value<0.0001

Data from 2015 revealed it was the hottest year in the past decade in Karachi. On average, the temperature of Karachi remains between 34°C to 36°C due to sea breeze but in June 2015 the temperature rose to 45°C and humidity dropped from 70% to 12%.^12^,^13^ Notably, the incidence of malaria was low in this year.

The incidence of malaria depended on humidity levels in the preceding month (Equation-1, Fig.2). The multiple negative binomial regression model suggested that if the humidity level was a unit higher in one month, cases will experience a statistically significant rise in the following month (Coeff: 0.0777, p=0.000, Equation-I). The year (trend) variable, shows visually the significantly positive effect on malaria cases, supporting secular incline in malaria cases (Equation-I). Also notable is the decline in malaria cases in 2015 related to the preceding month humidity.

**Equation-I:** Negative binomial regression model.

Log (Malaria) = intercept+ a*lag (mean humidity) + b*trend

Log (Malaria) = -1.853 + 0.0777 *lag (mean humidity) + 0.0399 *trend (AIC: 10.2)

(P-value=0.0080) (P-value=0.000) (P-value=0.000)

## DISCUSSION

Karachi is located along the Arabian coast and is the largest city of Pakistan. It experiences moderately high temperatures with high humidity.^14^ The rapidly increasing population and urbanization cause crowding, poverty, poor infrastructure and sanitation. All these features are conducive for the proliferation of mosquitoes.

We found temperature and humidity had a significant influence on malaria cases. This finding is consistent with other studies in which temperature is considered to be the precipitating factor for the Anopheles mosquito.^15^,^16^ In the Guangzhou area of China, rise in temperature and relative humidity has been shown to have a positive association with malaria incidence.^17^ Kipruto et al. established that rainfall had the most dominant cross correlation with malaria cases in riverine, highlands and lowlands regions and along with temperatures in highland and riverine contributed to variations in malaria incidence.^18^ Meanwhile, studies in Korea found sunshine to be positively associated with malaria incidence.^19^

In our study, one-unit increase in humidity of the previous month led to higher malaria cases in the incident month. This correlation of temperature and one month lag period with the monthly incidence of malaria cases was also observed in a study conducted over 12 years in the Shuchen county of China.^20^ Kipruto et al. also associated with a two month lag for rainfall in riverine, highlands and lowlands and up to one month for increase in temperature.^18^ The lag period in our case of one month compared with 0-4 months in other studies may be explained by the vector need for a fertile environment for its survival until it matures^21^ which may be geographically variable between these studies.

A study conducted in Peshawar, Pakistan had a total of 411 diagnosed malaria cases. Almost a third (32.60%) presented in the autumn (falciparum=66.42%), 9% in winter (falciparum=67.6%), 18.5% in spring (vivax=93.4%) and 40% in summer (vivax=89.6%). The authors concluded that malaria showed a highly significant pattern in different seasons of the year such that P. falciparum and P. vivax malaria reached their highest frequency in autumn and winter seasons and in spring and summer seasons respectively.^22^ The data on species correlation to seasonality could not be assessed in our study, due to half the data being missing. However, of those which were mentioned vivax was the predominant specie, which correlates with the 2018 world malaria report’s statistics for Pakistan.^3^

The decline noted in our study in 2015 is as per the World Report for Pakistan.^3^ This may be attributed to it being the hottest year in the past 10 years in Karachi as seen in our study and evidence that temperature change have altered vector behavior.^1^,^14^ This may influence incidence apart from the other measures taken for vector control. By understanding the seasonality of malaria parasite, it is easier to prepare and implement malaria control measures. The preparation should be done well ahead of the peak transmission period by targeting mosquito and infected people simultaneously. The findings of this study provide preliminary information that can be useful to develop malaria early warning system.

## CONCLUSION

The incidence of malaria presenting to TIH has risen over recent years and significant seasonal variations in Karachi particularly temperature and humidity, has contributed to this.

### Author`s Contribution

**FH:** Conceived the idea, designed the proposal, writing & editing of manuscript.

**SI:** Statistical analysis, results write-up, formatting & editing of manuscript.

**AN:** Data collection, literature review and manuscript writing.

**SR:** Manuscript writing.
